# Association between the C-Reactive Protein–Albumin–Lymphocyte (CALLY) Index and Adverse Clinical Outcomes in CAD Patients after PCI: Findings of a Real-World Study

**DOI:** 10.31083/j.rcm2504111

**Published:** 2024-03-25

**Authors:** Ying Pan, Ting-Ting Wu, Chang-Jiang Deng, Zhi-Hui Jiang, Yi Yang, Xian-Geng Hou, Tuo Yan, Shun Wang, Yu-Juan Feng, Ying-Ying Zheng, Xiang Xie

**Affiliations:** ^1^State Key Laboratory of Pathogenesis, Prevention, Treatment of Central Asian High Incidence Diseases, 830054 Urumqi, Xinjiang, China; ^2^Department of Cardiology, Xinjiang Medical University Affiliated First Hospital, 830054 Urumqi, Xinjiang, China

**Keywords:** CRP–albumin–lymphocyte index, coronary artery disease, percutaneous coronary intervention, prognosis, biomarker

## Abstract

**Background:**

The C-reactive protein–albumin–lymphocyte (CALLY) index is a novel inflammatory biomarker, and its association with the prognosis of coronary artery disease (CAD) after percutaneous coronary intervention (PCI) has not previously been studied. Therefore, this study aimed to investigate the effect of using the CALLY index on adverse outcomes in CAD patients undergoing PCI.

**Methods:**

From December 2016 to October 2021, we consecutively enrolled 15,250 CAD patients and performed follow-ups for primary endpoints consisting of all-cause mortality (ACM) and cardiac mortality (CM). The CALLY index was computed using the following formula: (albumin × lymphocyte)/(C-reactive protein (CRP) × ${\mathrm{10}}^{4}$). The average duration of the follow-up was 24 months.

**Results:**

A total of 3799 CAD patients who had undergone PCI were ultimately enrolled in the present study. The patients were divided into four groups according to the CALLY index quartiles: Q1 (≤0.69, n = 950), Q2 (0.69–2.44, n = 950), Q3 (2.44–9.52, n = 950), and Q4 (>9.52, n = 949). The low-Q1 group had a significantly higher prevalence of ACM (*p* < 0.001), CM (*p* < 0.001), major adverse cardiac events (MACEs) (*p* = 0.002), and major adverse cardiac and cerebrovascular events (MACCEs) (*p* = 0.002). Kaplan–Meier analysis revealed that a low CALLY index was significantly linked with adverse outcomes. After univariate and multivariate Cox regression analysis, the risk of ACM, CM, MACEs, and MACCEs decreased by 73.7% (adjust hazard risk [HR] = 0.263, 95% CI: 0.147–0.468, *p* < 0.001), 70.6% (adjust HR = 0.294, 95% CI: 0.150–0.579, *p* < 0. 001), 37.4% (adjust HR = 0.626, 95% CI: 0.422–0.929, *p* = 0.010), and 41.5% (adjust HR = 0.585, 95% CI: 0.401–0.856, *p* = 0.006), respectively, in the Q4 quartiles compared with the Q1 quartiles.

**Conclusions:**

This study revealed that a decreased CALLY index was associated with worse prognoses for CAD patients after PCI. The categorization of patients with a decreased CALLY index could provide valuable evidence for the risk stratification of adverse outcomes in CAD patients after PCI.

**Clinical Trial Registration:**

The details are available at http://www.chictr.org.cn (Identifier: NCT05174143).

## 1. Introduction

Since percutaneous coronary intervention (PCI) was introduced over five decades ago, the prognosis for patients with coronary artery disease (CAD) has improved significantly [1, 2]. However, despite great advancements in the treatment of CAD, the morbidity and mortality from cardiovascular diseases in China continue to rise [3]. Inflammation occurs during the occurrence and development of atherosclerosis and has an important impact on triggering cardiovascular diseases [4]. Several new humoral biomarkers of inflammation have been established to predict the long-term outcomes of CAD patients. However, few reports have verified the practical application value of these markers in daily diagnosis and treatment processes. Therefore, there is a need for the identification of novel biomarkers regarding the risk assessment of clinical outcomes in patients after PCI.

The C-reactive protein–albumin–lymphocyte (CALLY) index, which consists of the C-reactive protein (CRP), albumin, and lymphocytes, combines the markers of inflammation, immunity, and nutrition. Iida *et al*. [5] initially proposed the CALLY index and reported that a low CALLY index was related to poor survival in hepatocellular carcinoma patients after hepatectomy. However, the significance of the CALLY index as a predictor has since been discovered in different cancers [6, 7, 8, 9, 10], although few data have been reported on the relationship between the CALLY index and cardiovascular events. Considering that it is easy to access and has a high quality–price ratio, the CALLY index may provide doctors with useful evidence for risk stratification in the prognosis of CAD patients undergoing PCI. In addition, since inflammatory, immunological, and nutritional conditions are commonly reported to be highly involved in cardiovascular events [11, 12, 13, 14], it is sensible to assess the predictive performance of the CALLY index in patients with CAD. Thus, using a cohort of real-world patients, we aimed to evaluate the effect of the CALLY index on the risk of long-term outcomes in patients undergoing PCI.

## 2. Methods

### 2.1 Study Design and Population

**Fig. 1.**
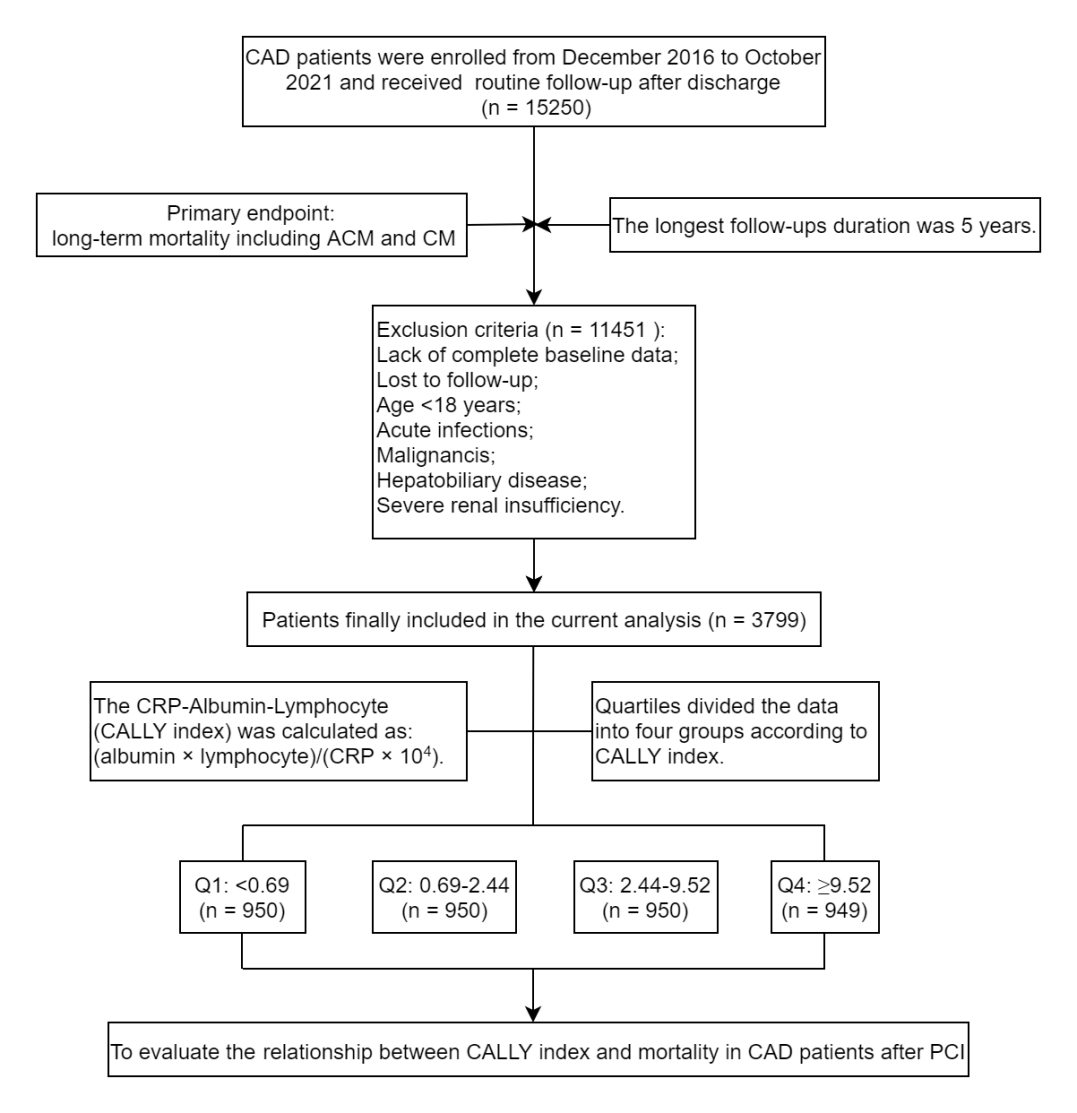
**Flowchart of the study**. CAD, coronary artery disease; PCI, percutaneous coronary intervention; CALLY, C-reactive protein–albumin–lymphocyte; ACM, all-cause mortality; CM, cardiac mortality; CRP, C-reactive protein.

From December 2016 to October 2021, 15,250 consecutive CAD patients were hospitalized at Xinjiang Medical University Affiliated First Hospital. These details are available at http://www.chictr.org.cn (Identifier: NCT05174143). Coronary artery disease was diagnosed as ≥50% stenosis of the left main vessel or ≥70% stenosis in at least one main vessel, as shown by coronary angiography. Patients in our study with PCI were those who had at least one stent successfully implanted by an experienced cardiologist. The study protocol complied with the Declaration of Helsinki and the Ethics Committee of Xinjiang Medical University Affiliated First Hospital. All subjects provided written informed consent prior to participation. A total of 11,451 patients were excluded due to either missing baseline and follow-up data, age <18 years, PCI failure, and/or other exclusion criteria (Fig. 1). The CALLY index was computed using the following formula: (albumin × lymphocyte)/(CRP × ${\mathrm{10}}^{4}$). Overall, 3799 CAD patients who had undergone PCI were enrolled in this analysis and assigned to four quartiles according to the CALLY index: Q1 (≤0.69, n = 950), Q2 (0.69–2.44, n = 950), Q3 (2.44–9.52, n = 950), and Q4 (>9.52, n = 949). We also analyzed the adverse outcomes in individuals with stable coronary artery disease (SCAD) (n = 1226) and acute coronary syndrome (ACS) (n = 2573).

### 2.2 Data Collection

Demographic data, cardiovascular risk factors, and laboratory data were documented for all patients. We recorded cardiovascular risk factors, such as sex, age, smoking status, drinking status, and history of hypertension and diabetes. We also collected information on medications. Fasting blood samples were collected within 24 h of admission and stored in –80 °C refrigerators until testing. Serum concentrations of creatinine (Cr), uric acid (UA), triglyceride (TG), total cholesterol (TC), high-density lipoprotein-C (HDL-C), low-density lipoprotein-C (LDL-C), lymphocyte count, albumin, and CRP were measured in the Clinical Laboratory Department of Xinjiang Medical University First Affiliated Hospital using chemical analysis equipment. The severity of the coronary artery stenosis was also collected in SCAD patients and ACS patients.

### 2.3 Follow-Up

Enrolled patients underwent follow-ups at 1 month, 6 months, 1 year, 3 years, and 5 years after discharge. The well-trained research coordinators evaluated the patients by office visits, telephone contact, or examination of medical records, as necessary. The occurrence of all-cause mortality (ACM) and cardiac mortality (CM) was the primary endpoint. The secondary endpoint was to assess major adverse cardiac events (MACEs), which is the combination of ACM, CM, non-fatal myocardial infarction, and unplanned coronary revascularization, alongside major adverse cardiac and cerebrovascular events (MACCEs), which were defined as MACEs plus stroke [15].

### 2.4 Statistical Analyses

Statistical analyses were performed using SPSS 26.0 (SPSS Inc., Chicago, IL, USA). Continuous variables are presented as either the mean (SD) or median (IQR). Categorical data are reported as numbers and percentages. We employed the *t*-test or analysis of variance and Chi-square tests or Fisher’s exact test to compare continuous and categorical variables, respectively. The Kaplan–Meier method was used to estimate the cumulative survival probabilities. Univariate and multivariate Cox regression models were used to assess hazard ratios (HRs) and corresponding 95% confidence intervals (CIs). Finally, we plotted the restricted cubic spline between the CALLY index and adverse clinical outcomes. A value of *p* < 0.05 was considered statistically significant.

## 3. Results

### 3.1 Baseline Characteristics

Table 1 displays the baseline characteristics of the cohort. Of the 3799 CAD participants who had undergone PCI, most patients were men (73.0%), while the mean age was 61.78 ± 11.99 years. No significant differences were observed for hypertension, TC, LDL-C, β-blockers, aspirin, statin, or clopidogrel use (*p* > 0.05) among the four groups. Several variables were significantly different, including sex, age, drinking, smoking, diabetes history, Cr, UA, HbA1c, TG, HDL-C, lymphocyte, albumin, CRP, angiotensin-converting enzyme inhibitor (ACEI), angiotensin receptor blocker (ARB), and proton pump inhibitors (PPI) (all *p* < 0.05). Notably, lymphocyte and albumin levels were lowest, while CRP was highest in Q1. In this cohort, there were 1226 SCAD individuals and 2573 ACS individuals. The baseline characteristics of these individuals are presented in **Supplementary Tables 1,2**. As shown in **Supplementary Table 1**, significant differences were only found for age and white blood cell (WBC) counts among the SCAD groups. However, among ACS patients, there were significant differences in sex, age, smoking, multivessel disease, WBC, TG, and HDL-C (**Supplementary Table 2**).

**Table 1.** **Baseline characteristics of participants**.

Variables	Q1	Q2	Q3	Q4
	(<0.69)	(0.69–2.44)	(2.44–9.52)	(≥9.52)
Male, n (%)	725 (76.3)	671 (70.6)	674 (70.9)	704 (74.2)
Age, mean (SD), years	63.52 ± 11.86	62.47 ± 11.88	60.86 ± 11.97	60.27 ± 11.98
Smoking, n (%)	411 (43.3)	373 (39.3)	348 (36.6)	353 (37.2)
Drinking, n (%)	244 (25.7)	217 (22.8)	203 (21.4)	192 (20.2)
Hypertension, n (%)	646 (68.0)	670 (70.5)	664 (69.9)	625 (65.9)
Diabetes, n (%)	686 (72.2)	630 (66.3)	534 (56.2)	501 (52.8)
SCAD, n (%)	258 (27.2)	287 (30.2)	318 (33.5)	363 (38.3)
SCr, median (IQR), µmol/L	78.7 [66.0–97.0]	74.0 [62.4–90.0]	72.1 [62.0–85.4]	73.0 [62.0–86.0]
UA, median (IQR), mmol/L	372.0 [299.0–482.3]	354.7 [295.8–448.2]	344.8 [287.1–416.7]	335.0 [280.0–403.0]
HbA1c, mean (SD), mmol/L	6.85 ± 1.65	6.90 ± 1.67	6.65 ± 1.48	6.45 ± 1.44
TG, median (IQR), mmol/L	1.2 [0.9–1.8]	1.4 [1.0–2.1]	1.6 [1.1–2.3]	1.5 [1.1–2.2]
TC, mean (SD), mmol/L	3.74 ± 1.12	3.86 ± 1.10	3.87 ± 1.06	3.84 ± 1.07
HDL-C, mean (SD), mmol/L	0.99 ± 0.30	1.01 ± 0.29	1.06 ± 0.33	1.09 ± 0.31
LDL-C, mean (SD), mmol/L	2.44 ± 0.90	2.50 ± 0.89	2.48 ± 0.88	2.44 ± 0.88
Lymphocytes, mean (SD), ×${\mathrm{10}}^{9}$/L	1.95 ± 0.82	2.19 ± 0.80	2.28 ± 0.85	2.36 ± 0.99
Albumin, mean (SD), g/L	37.77 ± 7.99	40.25 ± 8.09	42.16 ± 7.68	43.38 ± 8.54
CRP, median (IQR), g/L	28.6 [16.2–63.0]	6.5 [4.6–8.9]	1.8 [1.3–2.7]	0.3 [0.2–0.6]
Multivessel disease, n (%)	867 (91.3)	835 (87.9)	816 (85.9)	786 (82.8)
ACEI/ARB, n (%)	388 (40.8)	460 (48.4)	414 (43.6)	396 (41.7)
β-blockers, n (%)	509 (56.3)	559 (60.8)	521 (58.0)	492 (54.6)
Other lipid-lowering drugs, n (%)	540 (59.7)	649 (70.5)	625 (69.5)	634 (70.3)
Aspirin, n (%)	890 (93.7)	897 (94.4)	905 (95.3)	913 (96.2)
Statin, n (%)	855 (90.0)	856 (90.1)	866 (91.2)	883 (93.0)
Anticoagulation after PCI, n (%)	136 (14.3)	93 (9.8)	116 (12.2)	139 (14.6)
PPI, n (%)	72 (7.6)	42 (4.4)	47 (4.9)	35 (3.7)
Clopidogrel, n (%)	477 (50.2)	480 (50.5)	460 (48.4)	504 (53.1)

Note: SCr, serum creatinine; UA, uric acid; HbA1c, hemoglobin A1c; TG, triglycerides; TC, total cholesterol; HDL-C, high-density lipoprotein-C; LDL-C, low-density lipoprotein-C; ARB, angiotensin receptor blocker; ACEI, angiotensin-converting enzyme inhibitor; CRP, C-reactive protein; PPI, proton pump inhibitors; SCAD, stable coronary artery disease.

### 3.2 Clinical Outcomes

Among all patients, there were 216 cases of ACM during the follow-up (Table 2). The incidence of ACM in the Q1 quartile was 105 (11.1%), while Q2 was 65 (6.8%), Q3 was 24 (2.5%), and Q4 was 22 (2.3%). The incidence of ACM was lower in the Q3 and Q4 quartiles than in Q1 (*p* < 0.001). We also found that CM occurred in 165 patients: 78 (8.2%) in Q1, 53 (5.6%) in Q2, 18 (1.9%) in Q3, and 16 (1.7%) in Q4 (*p* < 0.001). Regarding the secondary endpoints, we found that MACEs and MACCEs occurred more frequently in Q1 patients (both *p* = 0.002). The occurrence of ACM and CM was similar among ACS patients. However, in SCAD patients, we found that patients in Q3 had the lowest rates of ACM and CM (*p* < 0.05), while the incidence of ischemic events, including MACEs and MACCEs, was not significantly different (*p* > 0.05).

**Table 2.** **Comparison of the outcomes in the four groups**.

Outcomes		Q1	Q2	Q3	Q4	Chi-square or *t*	*p*-value
		(<0.69)	(0.69–2.44)	(2.44–9.52)	(≥9.52)		
All patients (n = 3799)							
	ACM, *n *(%)	105 (11.1)	65 (6.8)	24 (2.5)	22 (2.3)	91.149	<0.001
	CM, *n *(%)	78 (8.2)	53 (5.6)	18 (1.9)	16 (1.7)	67.527	<0.001
	MACEs, *n *(%)	117 (12.3)	95 (10.0)	80 (8.4)	70 (7.4)	15.257	0.002
	MACCEs, *n *(%)	125 (13.2)	102 (10.7)	85 (8.9)	78 (8.2)	14.962	0.002
SCAD patients (n = 1226)							
	ACM, *n *(%)	25 (9.7)	15 (5.2)	6 (1.9)	9 (2.5)	25.098	<0.001
	CM, *n *(%)	13 (5.0)	14 (4.9)	3 (0.9)	5 (1.4)	15.720	0.001
	MACEs, *n *(%)	23 (8.9)	26 (9.1)	18 (5.7)	17 (4.7)	7.294	0.063
	MACCEs, *n *(%)	27 (10.5)	26 (9.1)	21 (6.6)	20 (5.5)	6.533	0.088
ACS patients (n = 2573)							
	ACM, *n *(%)	80 (11.6)	50 (7.5)	18 (2.8)	13 (2.2)	63.864	<0.001
	CM, *n *(%)	65 (9.4)	39 (5.9)	15 (2.4)	11 (1.9)	49.902	<0.001
	MACEs, *n *(%)	94 (13.6)	69 (10.4)	62 (9.8)	53 (9.0)	8.188	0.042
	MACCEs, *n *(%)	98 (14.2)	76 (11.5)	64 (10.1)	58 (9.9)	7.466	0.058

ACM, all-cause mortality; CM, cardiac mortality; MACEs, major adverse cardiovascular events; MACCEs, major adverse cardiac and cerebrovascular events; ACS, acute coronary syndrome; SCAD, stable coronary artery disease.

**Fig. 2.**
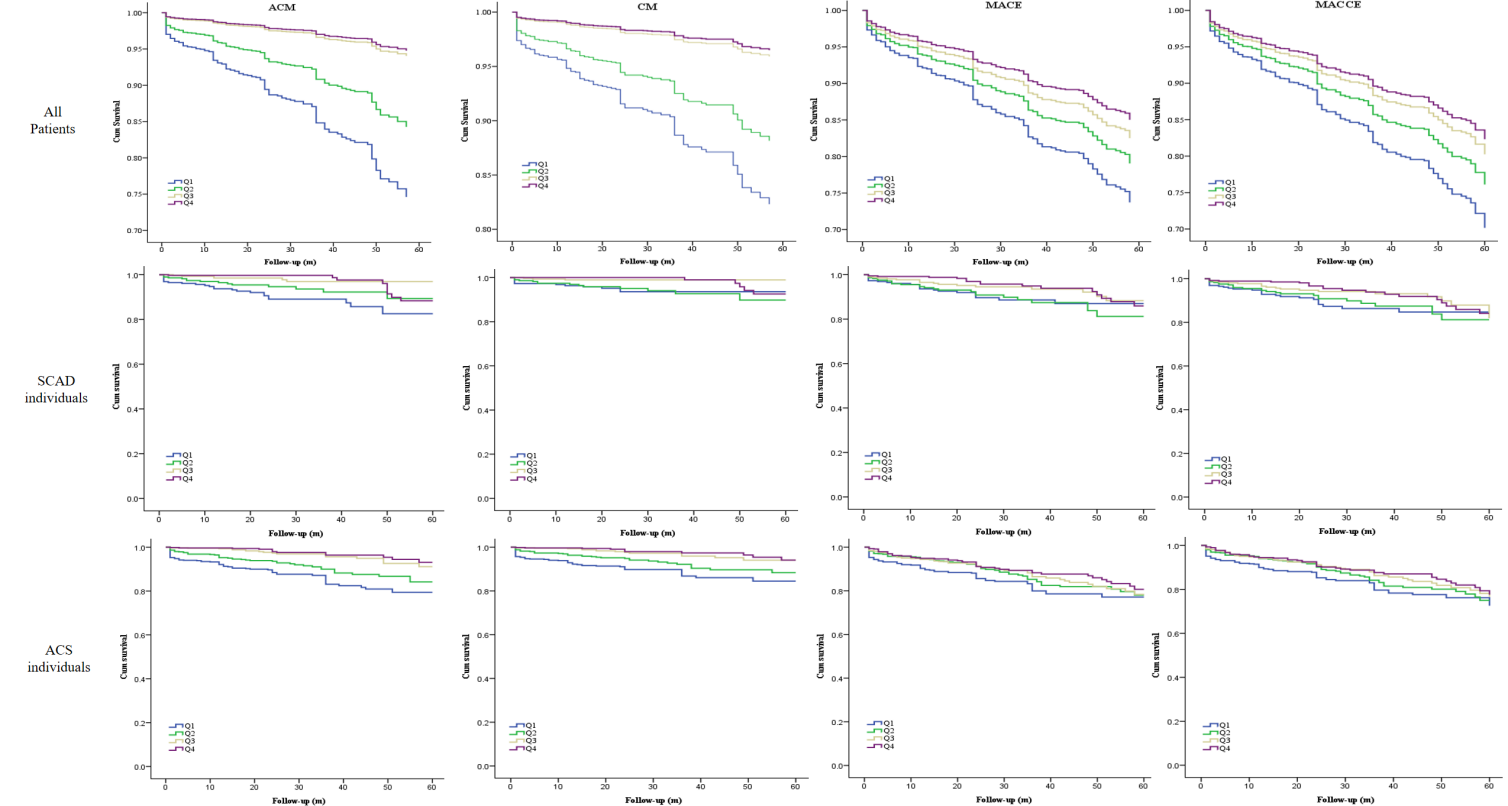
**Cumulative Kaplan–Meier estimates of the time for clinical outcomes to occur in all patients according to the CALLY index quartile**. ACM, all-cause mortality; CM, cardiac mortality; MACEs, major adverse cardiovascular events; MACCEs, major adverse cardiac and cerebrovascular events; SCAD, stable coronary artery disease; ACS, acute coronary syndrome; CALLY, C-reactive protein–albumin–lymphocyte.

Kaplan–Meier curves for the CALLY index and outcomes are shown in Fig. 2. In total, patients in high-CALLY quartiles (Q2, Q3, and Q4) showed a significantly decreased risk of ACM, CM, MACEs, and MACCEs compared with patients in the low-Q1 quartile.

**Fig. 3.**
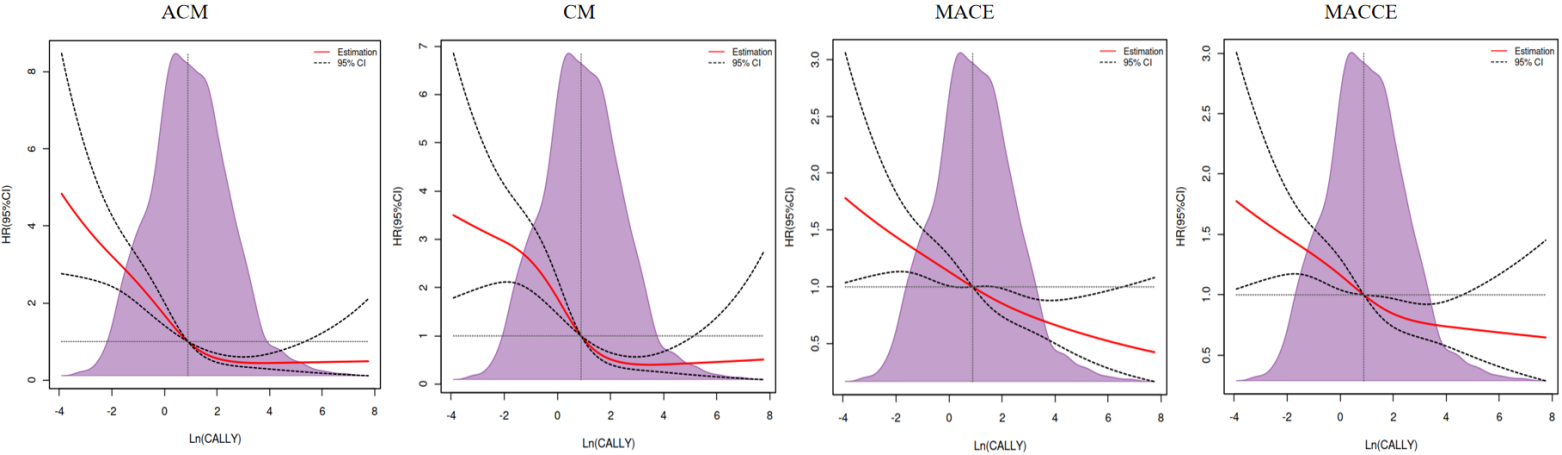
**Restricted cubic spline plots for mortality and ischemic events according to Ln (CALLY) continuous scale**. The shaded (light purple color) area represents the percentage of the density distribution in the study population after using the CALLY index. Solid red lines are multivariable-adjusted hazard ratios (HRs), with black dotted ribbons showing 95% confidence intervals (CIs) derived from restricted cubic spline regressions with four knots. The horizontal dotted lines represent an HR of 1.0. ACM, all-cause mortality; CM, cardiac mortality; MACEs, major adverse cardiovascular events; MACCEs, major adverse cardiac and cerebrovascular events; CALLY, C-reactive protein–albumin–lymphocyte.

Then, we performed univariate and multivariate regression analyses and found that the CALLY index has good predictive value for poor prognoses of CAD patients (Table 3). After adjusting for traditional risk factors, including the history of smoking and drinking, sex, age, Cr, UA, HbA1C, TG, and HDL-C, the risks of ACM, CM, MACEs, and MACCEs were decreased by 77.2% (HR: 0.228, 95% CI: 0.121–0.427, *p <* 0.001), 74.8% (HR: 0.252, 95% CI: 0.122–0.524, *p* < 0.001), 32.9% (HR: 0.671, 95 CI: 0.456–0.986, *p* = 0.042), and 36.1% (HR: 0.639, 95% CI: 0.442–0.924, *p* = 0.017), respectively, in Q3, and by 73.7% (HR: 0.263, 95% CI: 0.147–0.468, *p* < 0.001), 70.6% (HR: 0.294, 95% CI: 0.150–0.579, *p* < 0.001), 37.4% (HR: 0.626, 95% CI: 0.422–0.929, *p* = 0.010), and 41.5% (HR: 0.585, 95% CI: 0.401–0.856, *p* = 0.006), respectively, in Q4 compared to in Q1 (See Table 3). The details of the multivariate regression analysis are shown in **Supplementary Tables 3,4,5,6**. We also plotted RCS curves to adequately estimate the relative hazard ratios in the CAD populations, using Ln (CALLY) as an independent variable (Fig. 3).

**Table 3.** **Univariate and multivariate Cox regression analysis for ACM**.

Outcomes		HR	(95% CI)	*p*-value	adjusted HR	(95% CI)	*p*-value
ACM (Q1 as reference)							
	Q2	0.584	0.429–0.796	0.001	0.725	0.488–1.076	0.111
	Q3	0.209	0.134–0.326	<0.001	0.228	0.121–0.427	<0.001
	Q4	0.183	0.116–0.291	<0.001	0.263	0.147–0.468	<0.001
CM (Q1 as reference)							
	Q2	0.648	0.457–0.918	0.015	0.844	0.537–1.327	0.462
	Q3	0.214	0.128–0.358	<0.001	0.252	0.122–0.524	<0.001
	Q4	0.183	0.107–0.314	<0.001	0.294	0.150–0.579	<0.001
MACEs (Q1 as reference)							
	Q2	0.771	0.588–1.011	0.060	0.899	0.635–1.275	0.552
	Q3	0.630	0.474–0.838	0.001	0.671	0.456–0.986	0.042
	Q4	0.532	0.395–0.716	<0.001	0.626	0.422–0.929	0.020
MACCEs (Q1 as reference)							
	Q2	0.770	0.593–1.001	0.051	0.887	0.636–1.237	0.481
	Q3	0.621	0.471–0.818	0.001	0.639	0.442–0.924	0.017
	Q4	0.549	0.413–0.729	<0.001	0.585	0.401–0.856	0.006

ACM, all-cause mortality; CM, cardiac mortality; MACEs, major adverse cardiovascular events; MACCEs, major adverse cardiac and cerebrovascular events; HR, hazard ratio; CI, confidence interval.

## 4. Discussion

To our knowledge, this is the first real-world, prospective, observational cohort study to investigate the prognosis of CAD patients undergoing PCI using different CALLY index levels. We analyzed the CALLY index in 3799 CAD patients who underwent PCI. We found that patients with the lowest CALLY index had the highest incidence of mortality, MACEs, and MACCEs. There were differences in sex, age, drinking status, smoking status, diabetes history, Cr, UA, HbA1c, TG, HDL-C, lymphocytes, albumin, CRP, ACEI/ARB, and PPI among the four quartiles. Considering the influence of these confounding factors, we conducted a multivariate Cox regression analysis, in which, we found that a low CALLY index was associated with poor prognosis in CAD patients who had undergone PCI. Therefore, the results were reliable and could not be contingent.

However, the mechanisms of action between the CALLY index and poor outcomes after PCI remain unclear. We noticed that patients with the lowest CALLY index in our study had the highest CRP, lowest albumin, and lowest lymphocyte levels. Both CRP and albumin were produced mainly in the liver and could reflect the inflammation grade. However, their acute phase response to inflammation was the opposite, with CRP levels rising and albumin levels decreasing [16]. Thus, CRP appears to act as a downstream biomarker that provides a function of overall upstream cytokine activation. It directly affects vascular disease through the binding and activation of complement and plays an important role in triggering immunity in plaque deposition [17, 18]. In 1994, Liuzzo *et al*. [19] first underlined that a higher CRP could predict poor prognosis in ACS patients. Subsequently, several investigators have focused on the predictive value of CRP in the risk of cardiovascular disease and adverse outcomes after PCI [20, 21, 22], and have finally drawn conclusions similar to those presented by Liuzzo *et al*. [19]. Albumin is the predominant serum protein and is associated with nutrition status and inflammatory conditions. Albumin participates in many physiological processes, such as binding various compounds, maintaining the colloidal osmotic pressure, and decreasing platelet aggregation [23]. Hypoalbuminemia is usually considered to be caused by malnutrition, inflammation, or cachexia [24]. Previous studies have shown that a low serum albumin concentration is a risk factor for poor prognosis among patients with MI, heart failure, and CAD undergoing PCI [25, 26, 27]. Importantly, Wada *et al*. [28] indicated that low serum albumin and high CRP had a cooperative effect on increasing long-term ischemic risk in patients after PCI. Lastly, the lymphocytic count can reportedly be used as an early marker of physiologic “stress” and systemic failure, secondary to myocardial ischemia [29, 30]. Alternatively, low lymphocyte levels represent immunodeficiency status and could predict adverse outcomes in CAD patients [31, 32]. These findings were consistent with our results and provided theoretical and clinical support for our conclusions. Therefore, the CALLY index, based on CRP, albumin, and lymphocyte levels, is a powerful and effective prognostic biomarker for CAD patients who have undergone PCI.

## 5. Study Limitations

The limitations of our study should be mentioned. Firstly, we only collected baseline data on CRP, albumin, and lymphocyte levels at admission. No information on the effects of changes in the CALLY index with time is available. The effect of dynamic changes in the CALLY index cannot be analyzed. Secondly, this study had a single cohort design. Our results need to be confirmed in the future by a multicenter study. Finally, the mechanism of action between the CALLY index and outcomes after PCI also requires further study.

## 6. Conclusions

In conclusion, this study suggests that the baseline CALLY index can be used as a novel, powerful, and inexpensive prognostic biomarker for CAD patients after PCI. The categorization of patients with a decreased CALLY index could provide valuable evidence for the risk stratification of CAD patients after PCI. However, this needs further validation.

## Data Availability

Data of this study were available from the corresponding author upon request.

## Supplementary Material

Supplementary material associated with this article can be found, in the online version, at https://doi.org/10.31083/j.rcm2504111.
