# Supplementary material for: Association between the C-Reactive Protein–Albumin–Lymphocyte (CALLY) Index and Adverse Clinical Outcomes in CAD Patients after PCI: Findings of a Real-World Study
Source: Rev Cardiovasc Med. 2024 Mar 25;25(4):111. doi: 10.31083/j.rcm2504111 (PMC11264017; doi:10.31083/j.rcm2504111)
Supplement: Supplementary file 1 [file 2153-8174-25-4-111-s1.pdf]

Supplementary Table 1 Baseline characteristics of SCAD participants (n = 1226).

variables	Q1 (<0.69)	Q2 (0.69-2.44)	Q3 (2.44-9.52)	Q4 (≥9.52)
Male, *n*(%)	194(75.2)	189(65.9)	233(73.3)	260(71.6)
Age, mean (SD), years	64.02±11.87	64.50±10.99	62.13±11.62	61.63±11.75
Smoking, *n*(%)	114(44.2)	109(38.0)	127(39.9)	141(38.8)
Drinking, *n*(%)	69(26.7)	65(22.6)	81(25.5)	67(18.5)
Hypertension, *n*(%)	187(72.5)	221(77.0)	238(74.8)	243(66.9)
Diabetes, *n*(%)	171(66.3)	168(58.5)	179(56.3)	182(50.1)
SCr,median (IQR), μmol/l	79.0[66.0-96.9]	72.0[62.8-88.1]	72.0[62.0-85.0]	74.0[62.0-88.0]
UA,median (IQR), mmol/L	370.0[297.2-462.8]	349.0[288.6-430.0]	333.4[282.8-417.2]	329.0[273.0-389.5]
HbA1c, mean (SD), mmol/L	6.94±1.72	6.93±1.71	6.62±1.46	6.43±1.42
TG, median (IQR), mmol/L	1.2[0.9-1.8]	1.4[1.1-2.1]	1.6[1.2-2.2]	1.4[1.0-2.1]
TC, mean (SD), mmol/L	3.71±1.05	3.87±1.05	3.81±0.99	3.84±1.11
HDL-C, mean (SD), mmol/L	1.01±0.27	1.05±0.29	1.06±0.31	1.10±0.31
LDL-C, mean (SD), mmol/L	2.42±0.87	2.47±0.83	2.43±0.83	2.43±0.88
lymphocyte, mean (SD), $\times 10^9$/L	1.95±0.75	2.15±0.75	2.20±0.77	2.34±0.96
Albumin, mean (SD), g/L	38.38±6.77	41.10±8.42	42.37±7.18	44.71±10.03
CRP, median (IQR), g/L	23.8[15.3-53.1]	6.4[4.3-8.9]	1.8[1.3-2.8]	0.4[0.2-0.6]
multivessel disease, *n*(%)	227(88.0)	251(87.5)	269(84.6)	300(82.6)
ACEI/ARB, *n*(%)	103(39.9)	130(45.3)	131(41.2)	168(46.3)
β-blockers, *n*(%)	140(57.6)	167(59.2)	180(60.4)	193(55.1)
Other lipid-lowering drugs, *n*(%)	139(56.7)	192(68.1)	217(72.8)	250(71.4)
Aspirin, *n*(%)	250(96.9)	278(96.9)	314(98.7)	352(97.0)
Statin, *n*(%)	240(93.0)	261(90.9)	283(89.0)	339(93.4)
Anticoagulation after PCI, *n*(%)	92(13.3)	76(11.5)	75(11.9)	89(15.2)
PPI, *n*(%)	26(10.1)	9(3.1)	11(3.5)	16(4.4)
Clopidogrel, *n*(%)	138(53.5)	154(53.7)	158(49.7)	186(51.2)

Note: SCr, serum creatinine; UA, uric acid; HbA1c, haemoglobin A1c; TG, triglycerides; TC, total cholesterol; HDL-C, high-density lipoprotein-C; LDL-C, low-density lipoprotein-C; ARB, angiotensin receptor blocker; ACEI, angiotensin-converting enzyme inhibitor; CRP, C-reactive protein.

Supplementary Table 2 Baseline characteristics of ACS participants (n = 2573).

variables	Q1 (<0.69)	Q2 (0.69-2.44)	Q3 (2.44-9.52)	Q4 (≥9.52)
Male, *n* (%)	531(76.7)	482(72.7)	441(69.8)	444(75.8)
Age, mean (SD), years	63.33±11.86	61.58±12.14	60.22±12.10	59.43±12.05
Smoking, *n* (%)	297(42.9)	264(39.8)	221(35.0)	212(36.2)
Drinking, *n* (%)	175(25.3)	152(22.9)	122(19.3)	125(21.3)
Hypertension, *n* (%)	459(66.3)	449(67.7)	426(67.4)	382(65.2)
Diabetes, *n* (%)	515(74.4)	462(69.7)	355(56.2)	319(54.4)
SCr,median (IQR), µmol/l	66.0[78.1-97.0]	75.0[62.2-90.4]	72.4[61.1-85.7]	72.2[62.0-84.4]
UA,median (IQR), mmol/L	373.5[299.3-490.0]	356.5[300.0-453.7]	348.0[289.0-416.5]	340.5[281.0-411.0]
HbA1c, mean (SD), mmol/L	6.94±1.72	6.93±1.71	6.62±1.46	6.43±1.42
TG, median (IQR), mmol/L	1.2[0.9-1.8]	1.4[1.0-2.0]	1.5[1.0-2.3]	1.5[1.1-2.4]
TC, mean (SD), mmol/L	3.76±1.15	3.86±1.12	3.90±1.09	3.83±1.04
HDL-C, mean (SD), mmol/L	0.98±0.31	1.00±0.29	1.06±0.35	1.08±0.31
LDL-C, mean (SD), mmol/L	2.45±0.91	2.52±0.92	2.51±0.90	2.46±0.87
WBC, mean (SD), $\times 10^9$ /L	9.00±3.36	8.90±3.01	8.29±2.79	7.92±2.16
lymphocyte, mean (SD), $\times 10^9$/L	1.95±0.84	2.20±0.82	2.33±0.89	2.37±1.01
Albumin, mean (SD), g/L	37.55±8.39	39.89±7.93	42.06±7.92	42.55±7.35
CRP, median (IQR), g/L	32.0[16.5-65.7]	6.5[4.7-8.9]	1.8[1.3-2.7]	0.3[0.2-0.6]
multivessel disease, *n* (%)	640(92.5)	584(88.1)	547(86.6)	486(82.9)
ACEI/ARB, *n* (%)	285(41.2)	330(49.8)	283(44.8)	228(38.9)
β-blockers, *n* (%)	369(55.8)	392(61.4)	341(56.7)	299(54.3)
Other lipid-lowering drugs, *n*(%)	401(60.8)	457(71.6)	408(67.9)	384(69.6)
Aspirin, *n* (%)	640(92.5)	619(93.4)	591(93.5)	561(95.7)
Statin, *n* (%)	615(88.9)	595(89.7)	583(92.2)	544(92.8)
Anticoagulation after PCI, *n* (%)	92(13.3)	76(11.5)	75(11.9)	89(15.2)
PPI, *n* (%)	46(6.6)	33(5.0)	36(5.7)	19(3.2)
Clopidogrel, *n* (%)	339(49.0)	326(49.2)	302(47.8)	318(54.3)

Note: SCr, serum creatinine; UA, uric acid; HbA1c, haemoglobin A1c; TG, triglycerides; TC, total cholesterol; HDL-C, high-density lipoprotein-C; LDL-C, low-density lipoprotein-C; ARB, angiotensin receptor blocker; ACEI, angiotensin-converting enzyme inhibitor; CRP, C-reactive protein.

Supplementary Table 3 Multivariable Cox Regression Analysis Results for ACM

Variables	B	SE	Wald	P	HR(95%)
age	0.065	0.009	57.985	<0.001	1.068(1.050-1.086)
sex	0.184	0.221	0.696	0.404	1.202(0.780-1.853)
smoking	-0.259	0.231	1.257	0.262	0.771(0.490-1.214)
drinking	0.290	0.258	1.271	0.260	1.337(0.807-2.215)
Cr	0.000	0.000	0.291	0.589	1.000(1.000-1.001)
UA	0.000	0.000	0.271	0.603	1.000(1.000-1.000)
HbA1c	0.154	0.051	9.119	0.003	1.167(1.056-1.289)
TG	-0.189	0.112	2.864	0.091	0.828(0.665-1.030)
HDL-C	-0.546	0.325	2.824	0.093	0.579(0.306-1.095)
Q1(as reference)			35.114	<0.001	
Q2 vs Q1	-0.322	0.202	2.545	0.111	0.725(0.488-1.076)
Q3 vs Q1	-1.480	0.321	21.284	**<0.001**	0.228(0.121-0.427)
Q4 vs Q1	-1.337	0.294	20.622	**<0.001**	0.263(0.147-0.468)

Supplementary Table 4 Multivariable Cox Regression Analysis Results for CM

Variables	B	SE	Wald	P	HR(95%)
age	0.059	0.010	36.018	<0.001	1.061(1.041-1.082)
sex	-0.013	0.248	0.003	0.958	0.987(0.607-1.604)
smoking	-0.202	0.275	0.540	0.462	0.817(0.477-1.400)
drinking	0.245	0.305	0.648	0.421	1.278(0.703-2.322)
Cr	0.000	0.000	0.079	0.778	1.000(0.999-1.001)
UA	0.000	0.000	0.001	0.976	1.000(1.000-1.000)
HbA1c	0.204	0.056	13.404	0.000	1.226(1.099-1.368)
TG	-0.234	0.130	3.228	0.072	0.791(0.613-1.021)
HDL-C	-0.684	0.379	0.541	0.462	0.844(0.537-1.327)
Q1(as reference)			22.935	<0.001	
Q2 vs Q1	-0.170	0.231	0.541	0.462	0.844(0.537-1.327)
Q3 vs Q1	-1.377	0.373	13.651	<0.001	0.252(0.122-0.524)
Q4 vs Q1	-1.224	0.345	12.558	<0.001	0.294(0.150-0.579)

Supplementary Table 5 Multivariable Cox Regression Analysis Results for MACE

Variables	B	SE	Wald	P	HR(95%)
age	0.020	0.006	10.536	0.001	1.020(1.008-1.033)
sex	0.089	0.174	0.259	0.611	1.093(0.777-1.537)
smoking	-0.165	-0.179	0.847	0.357	0.848(0.597-1.205)
drinking	0.077	0.192	0.160	0.690	1.080(0.741-1.572)
Cr	0.000	0.000	0.995	0.319	1.000(1.000-1.001)
UA	0.000	0.000	0.516	0.472	1.000(1.000-1.000)
HbA1c	0.131	0.038	11.641	0.001	1.140(1.057-1.229)
TG	-0.073	0.063	1.331	0.249	0.930(0.822-1.052)
HDL-C	-0.374	0.256	2.144	0.143	0.688(0.417-1.135)
Q1(as reference)			7.545	0.056	
Q2 vs Q1	-0.106	0.178	0.355	0.552	0.899(0.635-1.275)
Q3 vs Q1	-0.399	0.197	4.121	**0.042**	0.671(0.456-0.986)
Q4 vs Q1	-0.468	0.202	5.398	**0.010**	0.626(0.422-0.929)

Supplementary Table 6 Multivariable Cox Regression Analysis Results for MACCE

Variables	B	SE	Wald	P	HR(95%)
age	0.017	0.006	8.735	0.003	1.018(1.006-1.029)
sex	0.119	0.166	0.514	0.474	1.126(0.813-1.560)
smoking	-0.155	0.171	0.821	0.365	0.857(0.613-1.197)
drinking	0.113	0.185	0.372	0.542	1.119(0.780-1.607)
Cr	0.000	0.000	0.767	0.381	1.000(1.000-1.001)
UA	0.000	0.000	0.766	0.381	1.000(1.000-1.000)
HbA1c	0.118	0.037	9.891	0.002	1.125(1.045-1.211)
TG	-0.092	0.063	2.100	0.147	0.913(0.806-1.033)
HDL-C	-0.117	0.237	0.243	0.622	0.890(0.560-1.415)
Q1(as reference)			10.574	0.014	
Q2 vs Q1	-0.119	0.170	0.496	0.481	0.887(0.636-1.237)
Q3 vs Q1	-0.447	0.188	5.674	**0.017**	0.639(0.442-0.924)
Q4 vs Q1	-0.535	0.194	7.643	**0.006**	0.585(0.401-0.856)
